# Risk of Atrial Fibrillation or Flutter Associated with Periodontitis: A Nationwide, Population-Based, Cohort Study

**DOI:** 10.1371/journal.pone.0165601

**Published:** 2016-10-31

**Authors:** Der-Yuan Chen, Ching-Heng Lin, Yi-Ming Chen, Hsin-Hua Chen

**Affiliations:** 1 Department of Medical Education, Taichung Veterans General Hospital, Taichung, Taiwan; 2 Department of Medical Research, Taichung Veterans General Hospital, Taichung, Taiwan; 3 Institute of Biomedical Science and Rong Hsing Research Center for Translational Medicine, Chung-Hsing University, Taichung, Taiwan; 4 School of Medicine, National Yang-Ming University, Taipei, Taiwan; 5 Division of Allergy, Immunology and Rheumatology, Department of Internal Medicine, Taichung Veterans General Hospital, Taichung, Taiwan; 6 School of Medicine, Chung-Shan Medical University, Taichung, Taiwan; 7 Institute of Public Health and Community Medicine Research Center, National Yang-Ming University, Taipei, Taiwan; University of Minnesota, UNITED STATES

## Abstract

**Objective:**

To investigate the risk of atrial fibrillation or atrial flutter in patients with periodontitis (PD) in comparison with individuals without PD.

**Methods:**

We used the 1999–2010 Taiwanese National Health Insurance Research Database to identify cases of PD in the year 2000 matching (1:1) with persons without PD during 1999–2000 according to sex and individual age as the control group. Using Cox proportional regression analysis adjusting for potential confounders, including age, sex, and comorbidities at baseline, and average annual number of ambulatory visits and dental scaling frequency during the follow-up period, we estimated hazard ratios (HRs) with 95% confidence intervals (CIs) to examine the risk of atrial fibrillation or flutter in PD patients in comparison with the control group. Subgroup analyses according to age, gender, or comorbidities were conducted to study the robustness of the association and investigate possible interaction effects.

**Results:**

We enrolled 393,745 patients with PD and 393,745 non-PD individuals. The incidence rates of atrial fibrillation or flutter were 200 per 10^5^ years among the PD group and 181 per 10^5^ years in the non-PD group (incidence rate ratio, 1.10; 95% CI, 1.06–1.14). After adjusting for potential confounders, we found an increased risk of atrial fibrillation or flutter in the PD group compared with the non-PD group (HR, 1.31; 95% CI, 1.25–1.36). The greater risk of atrial fibrillation or flutter in the PD group remained significant across all disease subgroups except hyperthyroidism and sleep apnea.

**Conclusion:**

The present study results indicate an increased risk of atrial fibrillation or flutter in patients with PD. Lack of individual information about alcohol consumption, obesity, and tobacco use was a major limitation.

## Introduction

Atrial fibrillation (AF) is the most common type of cardiac arrhythmia worldwide. The prevalence of AF is increasing in Western countries as well as in China [1–3]. In southwest China, about one in five adults experience AF in their lifetime [4]. The incidence of AF in the Taiwanese population aged ≥18 years is 1.5 per 1000 years [5]. AF may lead to harmful consequences, such as heart failure and thromboembolic diseases [6].

Inflammation, as well as atrial electrical and structural remodeling, is suggested to play a role in the initiation and perpetuation of AF [7–11]. Lymphomononuclear cell infiltration of the atrium has been demonstrated in patients with AF, but not among patients with normal sinus rhythm [12]. Systemic inflammation, characterized by serum inflammatory markers including tumor necrosis factor-α, interleukin (IL)-6, IL-2, and C-reactive protein, were associated with a higher risk of AF development [11, 13–15]. Previous studies have also shown that some chronic inflammatory diseases, such as rheumatoid arthritis and psoriasis, were associated with a higher risk of AF development [16–20]. One study showed that the use of corticosteroid, an anti-inflammatory agent, decreased the risk of AF recurrence after electrical cardioversion [21]. Furthermore, in a meta-analysis of 50 randomized controled trials, prophylactic corticosteroids reduced AF risk among patients undergoing cardiac surgery compared with controls [22]. In a canine sterile pericarditis animal model, corticosteroid therapy also attenuated the incidence of inducible AF [23]. Taken together, it is reasonable to hypothesize that systemic inflammation may lead to the development of AF [7].

Periodontitis (PD) is a common periodontal inflammatory disorder initiated by bacteria, such as *Porphyromonas gingivalis (P*.*gingivalis)*, *Tannerella forsythia*, and *Treponema denticola*. PD may result in the destruction of the supporting tissue of the teeth, eventually resulting in tooth loss if not treated [24]. Systemic inflammation markers have been found to be elevated in patients with PD [25–30]. Possible mechanisms linking PD to systemic inflammation included: 1) the entry of locally produced inflammatory cytokines into the circulation, inducing an acute-phase response in the liver; 2) the systemic dissemination of periodontal bacteria via gingival ulcerations in periodontal pockets; and 3) increased gut epithelial permeability and endotoxaemia caused by gut microbiota alterations due to swallowed *P*.*gingivalis* [31]. Additionally, serum inflammatory markers decreased after periodontal therapy in PD patients [32]. Recently, an animal study using 22 adult mongrel canines of both sexes demonstrated that PD may lead to an inflammatory response in the atrial myocardium, facilitating the development of AF [33]. A nationwide, population-based cohort study showed that dental scaling, which may reduce the risk of PD, was associated with a lower risk of AF [34]. However, a cross-sectional study did not reveal an association between PD and cardiac arrhythmias in community-residing individuals aged ≥80 years [35]. However, to our knowledge, no previous longitudinal studies have investigated the association between PD and the risk of AF.

The Taiwanese National Health Insurance Research Database (NHIRD) has facilitated many population-based longitudinal studies. Therefore, we investigated the risk of AF or atrial flutter (AFL) associated with PD by estimating the hazard ratio (HR) for AF or AFL in cases with PD compared with a control group without PD using claims data from the NHIRD.

## Methods

### Ethics statement

The Ethics Committee for Clinical Research of Taichung Veterans General Hospital (Taichung, Taiwan) approved this study. Since the dataset detached all personal identification information before analysis, written informed consent was not attained.

### Study design

This study used a retrospective cohort study design.

### Data source

The NHIRD is a nationwide, single-payer, obligatory health insurance program launched on March 1, 1995, covering over 99% of the Taiwanese population. The National Health Research Institute maintains the NHIRD. The NHIRD is released for research purposes and consists of comprehensive claims data, including dental services, hospitalization services, outpatient serivces, traditional Chinese medical services, and detailed drug prescription records. However, the NHIRD lacks personal information, such as smoking and drinking, and results of laboratory tests, imaging, and pathology. The accuracy of the data has been improved by regular checks of patient medical charts by the Bureau of the National Health Institute [36].

The present study used 1999–2010 enrollment, inpatient,ambulatory care, dental service, and prescription drug records of the NHIRD. We used these comprehensive data to create a nationwide PD cohort group selected in 2000. The NHIRD also built a longitudinal health insurance database of 1,000,000 individuals randomly chosen from all enrollees who were included in the 2000 enrollment files (LHID2000). The data of the control group were obtained from LHID2000.

### Definition of PD

The Taiwanese government encourages people to receive regular dental scaling (DS) every six months to enhance dental care. However, if no PD is detected, more than two DS are not covered during one year. If a patient received a routine DS at a regular dental check-up and did not have PD, a dentist might still code a diagnosis of PD (ICD9-CM Codes 523.3–5). Therefore, the present study defined PD as having at least one dental visit recorded with PD (ICD9-CM Codes 523.3–5) and receiving antibiotic therapy or periodontal treatment simultaneously, or having more than two dental visits with a PD diagnosis and concurrent DS within one year.

### Study subjects

#### PD cohort selected from whole Taiwanese population

We enrolled all Taiwanese patients fitting the definition of PD in 2000 as the PD cohort. Those who had diagnoses of AF or AFL (ICD9-CM Codes 427.31–2) before 2000 were excluded.

#### Non-PD cohort selected from representative one million population

Because some individuals with periodontal disease other than PD might had undiagnosed subclinical PD, we selected all individuals without diagnosis of any periodontal disease (ICD9-CM Codes 523.x) during the period of 1999–2000 as the non-PD cohort from the LHID2000. We then matched the PD cohort with the non-PD cohort with a 1:1 ratio based on sex and individual age at baseline.

### Index dates

We defined the index date as the initial day of the study period. For the PD cohort, this study used the date of the first ambulatory visit at which the PD diagnosis was made (ICD9-CM Codes 523.3–5) in 2000. For the non-PD cohort, we used January 1, 2000, as the index date.

### Definition of incident AF or AFL

Incident AF or AFL was defined as having at least one outpatient or inpatient diagnosis of AF/AFL (ICD9-CM Codes 427.31–2) after the index date, and no history of an ambulatory visit with an AF/AFL diagnosis before the index date.

### Outcome variable

The outcome variable was the time from the index date to the date of the first outpatient or inpatient visit with a diagnosis of AF/AFL (ICD9-CM Codes 427.31–2). The censoring time was denoted as the date of withdrawal from the National Health Insurance for any reason, or December 31, 2010, whichever came first. If individuals in the control group developed PD after December 31, 2000, and before the date of the first ambulatory visit with a diagnosis of AF/AFL during the follow-up period, the date of withdrawal from the National Health Insurance for any reason, and December 31, 2010, the first date of PD diagnosis was used as the end of follow-up.

### Covariates

Potential confounders used for adjustment in the Cox regression analyses include age (<65 years or ≥65 years), sex, and comorbidities at baseline and average annual number of outpatient visits and dental scaling frequency (0, >0 and ≤2, >2) during the follow-up period. Cormorbidies included heart failure (ICD9-CM Code 428), hypertension (ICD9-CM Codes 401–5), diabetes mellitus (DM) (ICD9-CM Code 250), vascular disease (ICD9-CM Code 410, 412, 440–445), hyperlipidemia (ICD9-CM Code 272), ischemic heart disease (ICD9-CM Code 411, 413, 414), valvular heart disease (ICD9-CM Codes 093.2, 394–397, 424, 746.3–746.6), chronic obstructive pulmonary disease (COPD) (ICD9-CM 490–3, 496), sleep apnea (ICD9-CM 780.51, 780.53, 780.57), renal disease (ICD9-CM Codes 580–7), hypothyroidism (ICD9-CM 243–4), and hyperthyroidism (ICD9-CM Code 242). The presence of a comorbidity was defined as having at least three outpatient visits or one inpatient visit with the corresponding diagnosis within six months before or after the index date. Average annual number of outpatient visits during the follow-up period was adjusted because it was associated with detection bias (i.e., more frequent ambulatory visits leading to higher chance of detecting AF/AFL). Dental scaling frequency during the observation period was selected as a covariate because it was associated with the risk of AF/AFL [34] and was unequally distributed between the groups.

### Statistical analyses

We used a two-sample t-test to compare continuous variables between the two groups and the chi-square test to compare categorical variables. We calculated the cumulative incidence of AF/AFL by the Kaplan—Meier estimate. We compared the cumulative incidence of AF/AFL between the PD group and the non-PD group using the log-rank test. We utilized a Cox proportional hazard regression model to estimate AF/AFL risk associated with PD status, shown as hazard ratios (HRs) with 95% confidence intervals (CIs). To determine the significance of the modification effects of gender, age, or comorbidies on the association between PD and AF/AFL risk, we used the Wald test to estimate the *P*-value of the coefficient related to the product of the covariate and PD. We defined the statistical significance using a two-tailed *P*-value of <0.05. We performed statistical analyses using SPSS version 20.0 for Windows (SPSS, IL, Chicago, USA).

## Results

We identified a total of 460,597 patients in the PD group and 668,101 individuals in the non-PD group. The demographic data and clinical characteristics of these two groups were shown in S1 Table. After matching these two groups with a 1:1 ratio according to baseline age and sex, we finally enrolled 393,745 subjects in both groups. Table 1 compares the demographic data and comorbidities between the two enrolled groups. The proportions of all comorbidities except heart failure were higher in the PD group compared with the non-PD cohort. During the follow-up period, the average annual number of outpatient visits and dental scaling frequency were higher in the PD group compared with the non-PD group.

**Table 1:** Demographic data and clinical characteristics of the study subjects.

	PD (n = 393,745)	Non-PD (n = 393,745)	*P*-value
Age (mean ± SD)	42 ± 17	42 ± 17	1.000
Age group [n (%)]			1.000
<65 years	341,554 (86.7)	341,554 (86.7)	
≥65 years	52,191 (13.3)	52,191 (13.3)	
Gender			1.000
Female	199,250 (50.6)	199,250 (50.6)	
Male	194,495 (49.4)	194,495 (49.4)	
Comorbidity			
Heart failure	1,887 (0.5)	1,974 (0.5)	0.160
Hypertension	41,654 (10.6)	30,933 (7.9)	<0.001
Diabetes mellitus	22,136 (5.6)	14,119 (3.6)	<0.001
Vascular disease	2,669 (0.7)	1,925 (0.5)	<0.001
Hyperlipidemia	13,035 (3.3)	7,125 (1.8)	<0.001
Ischemic heart disease	13,231 (3.4)	8,624 (2.2)	<0.001
Valvular heart disease	2,256 (0.6)	1,481 (0.4)	<0.001
COPD	13,168 (3.3)	11,581 (2.9)	<0.001
Renal disease	4,023 (1.0)	3,520 (0.9)	<0.001
Hyperthyroidism	1,744 (0.4)	1,033 (0.3)	<0.001
Hypothyroidism	649 (0.2)	342 (0.1)	
Sleep apnea	124 (0.0)	49 (0.0)	<0.001
Average annual number of outpatient visits*	18 ± 15	13 ± 13	<0.001
Group of average annual number of outpatient visits*			<0.001
≤12	156,986 (39.9)	233,558 (59.3)	
>12	236,759 (60.1)	160,187 (40.7)	
Dental scaling frequency, number per year*	0.5 ± 0.6	0.1 ± 0.2	<0.001
Group of dental scaling frequency, number per year*			<0.001
None	38,166 (9.7)	221,175 (56.2)	
0 < scaling number ≤ 2	354,379 (90.9)	172,569 (43.8)	
2 < scaling number	1,200 (0.3)	1 (0.0)	

Abbreviations: PD, periodontitis; SD, standard deviation; COPD, chronic obstructive pulmonary disease.

*Calculated during the follow-up period.

**Fig 1:**
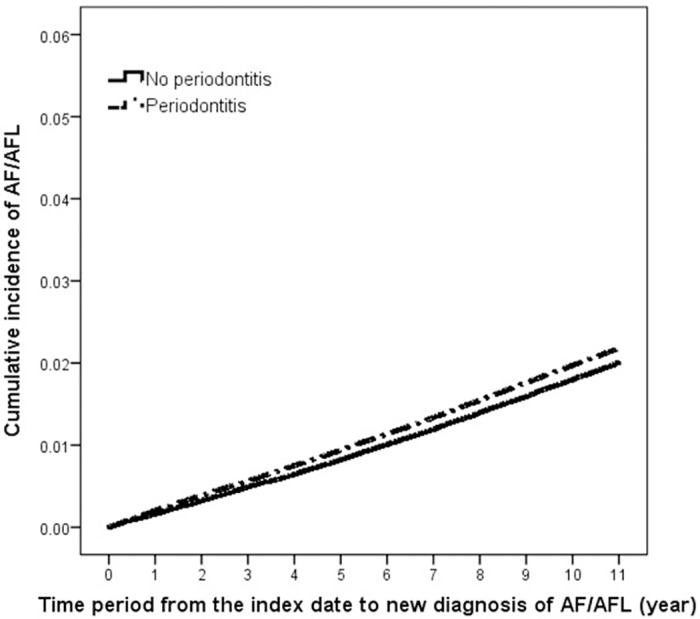
Cumulative incidence of atrial fibrillation or flutter rates in patients with periodontitis and in individuals without periodontitis.

As shown in Table 2, 8,138 (2.07%) of 393,745 PD patients developed AF/AFL during 4,075,682 person-years of follow-up. Among non-PD individuals, 6,180 (1.57%) had incident AF/AFL during 3,405,292 person-years of follow-up. The incidence rates of AF/AFL were 200 per 10^5^ years in the PD cohort and 181 per 10^5^ years in the non-PD cohort. The crude incidence rate ratio (IRR) of AF/AFL in the PD cohort compared with the non-PD cohort was 1.10 (95% CI, 1.06–1.14). Fig 1 shows the cumuative incidence of AF/AFL in the PD and non-PD cohorts during the follow-up period using the Kaplan—Meier method. The log-rank test showed that the cumulative incidence of AF/AFL was significantly higher in the PD group than that in the non-PD group (*P* < 0.001).

**Table 2:** Incidence rates of atrial fibrillation and flutter according to periodontitis status.

Variable	Total	Event (%)	Total person-years	Incidence rate (/10^5^ years)	IRR (95% CI)
Periodontitis status					
No periodontitis	393,745	6,180 (1.57)	3,405,292	181	1
Periodontitis	393,745	8,138 (2.07)	4,075,682	200	1.10 (1.06–1.14)

Table 3 shows the risk of AF/AFL associated with each covariate using univariate and multivariate (adjusting for all covariates) Cox regression analyses. In the univariate analysis, the risk of AF/AFL was higher in the PD cohort than that in the non-PD cohort (HR, 1.09; 95% CI, 1.06–1.13). After adjusting for potential confounders, the risk of AF/AFL in the PD cohort remained significantly higher than that in the non-PD cohort (HR, 1.31; 95% CI, 1.25–1.36). Other significant risk factors for the development of AF/AFL included age≥65 years, male gender, average annual number of outpatient visits >12, average annual number of dental scaling >2, heart failure, hypertension, DM, ischemic heart disease, valvular heart disease, COPD, renal disease, and hyperthyroidism. Individuals who received average annual dental scaling ≤2 had a lower risk of AF/AFL compared with those who never received dental scaling during the follow-up period.

**Table 3:** Univariate and multivariate analyses of the risk of atrial fibrillation or flutter associated with each variable shown as hazard ratio with 95% confidence intervals.

	Univariate analysis	Multivarate analysis
Periodontitis status		
No periodontitis	Reference	Reference
Periodontitis	1.09 (1.06–1.13)	1.31 (1.25–1.36)
Age group		
<65 years	Reference	Reference
≥65 years	12.52 (12.10–12.95)	5.56 (5.34–5.78)
Gender		
Female	Reference	Reference
Male	1.45 (1.40–1.49)	1.42 (1.37–1.47)
Average anual number of outpatient visits		
≤12	Reference	Reference
>12	5.53 (5.28–5.79)	3.23 (3.08–3.40)
Dental scaling frequency, number per year		
None	Reference	Reference
0 < scaling number ≤ 2	0.41 (0.40–0.43)	0.39 (0.38–0.41)
2 < scaling number	11.11 (9.91–12.46)	6.06 (5.38–6.83)
Comorbidities		
Heart failure	15.18 (14.08–16.36)	2.18 (2.01–2.37)
Hypertension	6.68 (6.45–6.91)	1.55 (1.49–1.61)
Diabetes mellitus	3.81 (3.64–4.00)	1.10 (1.04–1.15)
Vascular disease	6.07 (5.51–6.68)	1.08 (0.98–1.20)
Hyperlipidemia	3.16 (2.96–3.36)	1.01 (0.94–1.07)
Ischemic heart disease	8.44 (8.08–8.82)	1.57 (1.49–1.64)
Valvular heart disease	11.05 (10.19–11.98)	2.44 (2.24–2.66)
Chronic obstructive pulmonary disease	5.65 (5.38–5.92)	1.37 (1.30–1.44)
Renal disease	4.95 (4.54–5.40)	1.34 (1.22–1.46)
Hyperthyroidism	2.56 (2.16–3.05)	2.41 (2.03–2.88)
Hypothyroidism	1.89 (1.35–2.65)	0.84 (0.60–1.18)
Sleep apnea	2.96 (1.54–5.68)	1.26 (0.66–2.43)

**Fig 2:**
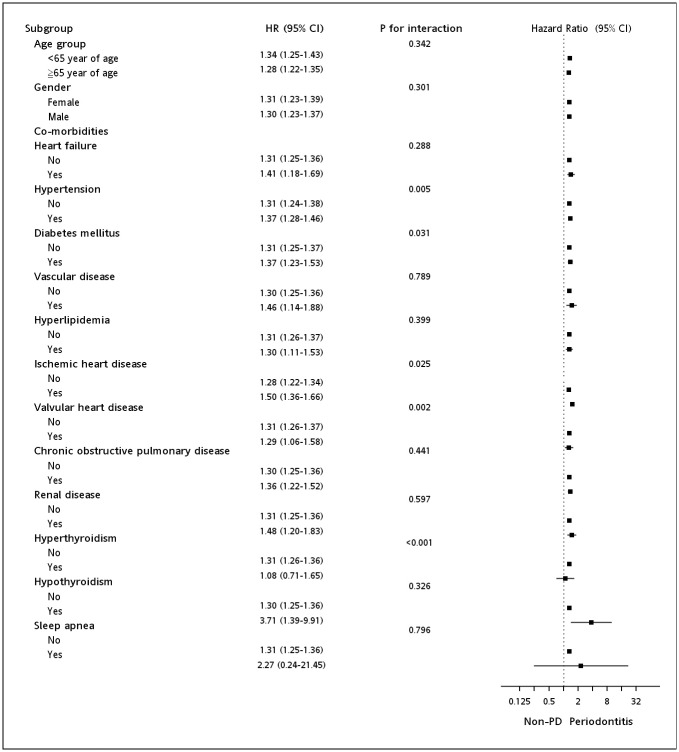
Risk of atrial fibrillation or flutter associated with periodontitis in the subgroups as stratified by other covariates. Adjusted variables included age, gender, average annual number of outpatient visits, and dental scaling frequency during the observation period and comorbidities.

Fig 2 shows the risk of AF/AFL associated with PD exposure among the various subgroups according to the baseline demographic data and comorbidities. The increased risk of AF or AFL in the PD group remained statistically significant across the subgroups, except hyperthyroidism and sleep apnea. The significance of modification effects by each covariate on the risk of AF/AFL associated with PD was also tested and the results are shown as *P* for interactions. Hypertension, DM, ischemic heart disease, valvular heart disease, and hyperthyroidism significantly modified the influence of PD on AF/ALF risk.

## Discussion

To our knowledge, our study is the first to assess the risk of AF/AFL asssociated with PD using a population-based cohort. The results show that patients with PD had a 31% higher risk of AF/AFL development than those without PD, after adjusting for measured potential confounders. This finding may be consistent with an animal study which demonstrated that PD induced AF development via the inflammatory process. Although inflammation has been found to be associated with the development, recurrence, persistence, or severity of PD [37–41], no prior longitudinal human studies have reported an association between PD, a chronic inflammatory disease, and AF/AFL.

Additionally, in this study, the positive association between PD exposure and AF/AFL risk remained statistically significant across the subgroups stratified by age (<65 years or ≥65 years), gender, heart failure, hypertension, DM, vascular disease, hyperlipidemia, ischemic heart disease, valvular heart disease, COPD, renal disease, and hypothyroidism. However, among patients with hyperthyroidism and sleep apnea, these associations did not reach statistical significance. One possible explanation might be inadequate statistical power due to the small case number for some of the diseases tested. Among patients with hyperthyroidism, another possible explanation is that this disease plays such a significant role in triggering the development of AF/ALF (HR, 2.41; 95% CI, 2.03–2.88) that it may mask the potential influence of PD. Therfore, the role of PD could only be revealed among the population without hyperthyroidism or sleep apnea. Consistent with a prior study [35], we did not find an increased risk of AF/AFL in PD patients aged 80 years and older (HR, 1.07; 0.95–1.21). This finding may be explained by the fact that older age (≥80 years) is a strong competing risk factor for AF/AFL development.

We also found that DS no more than twice per year was associated with a lower risk of AF/AFL, consistent with a prior study [34]. This finding may be explained by the protective effect for PD by DS. However, DS more than twice per year was associated with an increased the risk of AF/AFL. Although Taiwanese people are encouraged to receive regular DS every 6 months to enhance dental care, the Bureau of the National Health Institute does not reimburse DS more than twice per year. Therefore, those who received DS more than twice per year might actually develop PD during the follow-up period, leading to an increased risk of AF/AFL.

Consistent with the results of our study, prior studies have also revealed associations of aging [42], male gender [43–46], heart failure [47, 48], hypertension [49, 50], DM [51, 52], ischemic heart disease [53–56], valvular heart disease [47, 57, 58], COPD [59, 60], chronic kidney disease [61, 62], and hyperthyroidism [63–65] with AF/AFL. Although a recent study revealed a possible association of subclinical hypothyroidism with AF/AFL [66], our study, consistent with another study using the Framingham Heart Study participants [67], did not show such an association. Inconsistent with previous studies [68, 69], we did not find a significant association between hypothyroidism or sleep apnea and AF. Such inconsistency may be due to the small case number of sleep apnnea in our study, providing inadequate statisitcal power to exhibit a significant association of sleep apnea with AF/AFL.

The strength of our study was minimizing the selection bias by utilizing a nationwide database of the Taiwanese population. Therefore, the results could be applied to the general population in Taiwan. In addition, the cohort study design shows a temporal association between PD exposure and AF/AFL, suggesting a possible causal relationship. Finally, the adjustment of average annual number of outpatient visits during the follow-up period reduced the detection bias.

However, this study also has some limitations. The first major limitation was the lack of information in the database about established or potential risk factors for AF, such as obesity, alcohol consumption, and tobacco use [50]. Thus, the validity of estimating the risk of AF/AFL associated with PD may be hampered since these risk factors are potential confounders. Second, the NHIRD lacked clinical data to validate the diagnoses of AF and AFL. Although we cannot avoid a misclassification bias related to AF/AFL diagnosis, this non-differential misclassification bias always drives our results toward the null, resulting in conservative estimates. Third, the accuracy of the diagnosis of PD, which was based on claims data, may be of concern. However, the Bureau of the National Health Institute enhanced the accuracy of diagnoses by regular checks of original medical records [36]. Fourth, the lack of information to assess the severity of PD precluded us from further investigation of the dose—response relationship between PD exposure and the risk of AF or AFL.

### Conclusion

This population-based cohort study is the first to suggest an association between PD exposure and the development of AF or AFL. Further prospective cohort studies with more comprehensive clinical data are warranted to provide further evidence for this relationship.

## Supporting Information

S1 Data Data of the PD and non-PD cohorts. (RAR)

S1 Table Demographic data and clinical characteristics of subjects. (DOCX)
