# Supplementary material for: Risk of Atrial Fibrillation or Flutter Associated with Periodontitis: A Nationwide, Population-Based, Cohort Study
Source: PLoS One. 2016 Oct 31;11(10):e0165601. doi: 10.1371/journal.pone.0165601 (PMC5087888; doi:10.1371/journal.pone.0165601)
Supplement: S1 Table — (DOCX) [file pone.0165601.s002.docx]

**Risk of Atrial Fibrillation or Flutter Associated with Periodontitis: a Nationwide, Population-based, Cohort Study**

**Authors:** Der-Yuan Chen, Ching-Heng Lin, Yi-Ming Chen, Hsin-Hua Chen*

**Supplemental materials**

Supplemental Table S1. Demographic data and clinical characteristics of subjects.

|  | Non-PD (n =668,101) |  | PD (n =460,597) | *P*-value |
| --- | --- | --- | --- | --- |
| Age, years (mean ± SD) | 30 ± 21 |  | 44 ± 16 | <0.001 |
| Age group |  |  |  | <0.001 |
| <65 years | 610,180 (91.3) |  | 391,531 (85.0) |  |
| ≥65 years | 57,921 (8.7) |  | 69,066 (15.0) |  |
| Gender |  |  |  | <0.001 |
| Female | 314,293 (47.0) |  | 223,101 (48.4) |  |
| Male | 353,808 (53.0) |  | 237,496 (51.6) |  |
| Average annual number of outpatient visits* | 12 ± 12 |  | 19 ± 16 | <0.001 |
| Group of average annual number of outpatient visits* |  |  |  | <0.001 |
| ≤12 | 416,257 (62.3) |  | 175,524 (38.1) |  |
| >12 | 251,844 (37.7) |  | 285,073 (61.9) |  |
| Dental scaling frequency, number per year* | 0.1 ± 0.2 |  | 0.5 ± 0.7 | <0.001 |
| Group of dental scaling frequency, number per year* |  |  |  | <0.001 |
| None | 392,477 (58.8) |  | 45,742 (9.9) |  |
| 0 < scaling number ≤ 2 | 275,623 (41.2) |  | 413,388 (89.8) |  |
| 2 < scaling number | 1 (0.0) |  | 1,467 (0.3) |  |
| Comorbidities |  |  |  |  |
| Heart failure | 2,312 (0.4) |  | 2,427 (0.5) | <0.001 |
| Hypertension | 32,830 (4.9) |  | 55,571 (12.1) | <0.001 |
| Diabetes mellitus | 14,860 (2.2) |  | 29,399 (6.4) | <0.001 |
| Vascular disease | 2,084 (0.3) |  | 3,546 (0.8) | <0.001 |
| Hyperlipidemia | 7,394 (1.1) |  | 17,227 (3.7) | <0.001 |
| Ischemic heart disease | 9,311 (1.4) |  | 17,626 (3.8) | <0.001 |
| Valvular heart disease | 1,714 (0.3) |  | 2,842 (0.6) | <0.001 |
| COPD | 18,221 (2.7) |  | 17,126 (3.7) | <0.001 |
| Renal disease | 4,080 (0.6) |  | 5,161 (1.1) | <0.001 |
| Hyperthyroidism | 1,179 (0.2) |  | 2,013 (0.4) | <0.001 |
| Hypothyroidism | 456 (0.1) |  | 796 (0.2) | <0.001 |
| Sleep apnea | 68 (0.0) |  | 155 (0.0) | <0.001 |
| Abbreviations: PD, periodontitis; SD, standard deviation; COPD, chronic obstructive pulmonary disease.  *Calculated during the follow-up period. | | | | |
